# Pear flower and leaf microbiome dynamics during the naturally occurring spread of *Erwinia amylovora*

**DOI:** 10.1128/msphere.00011-25

**Published:** 2025-05-05

**Authors:** Aia Oz, Orly Mairesse, Shira Raikin, Hila Hanani, Hadar Mor, Mery Dafny Yelin, Itai Sharon

**Affiliations:** 1 Migal–Galilee Technology Center, Kiryat Shmona, Israel; 2 Northern Agriculture Research & Development, Migal–Galilee Technology Center, Kiryat Shemona, Israel; 3 Faculty of Sciences and Technology, Tel-Hai Academic College 54625, Upper Galilee, North District, Israel; University of Michigan, Ann Arbor, Michigan, USA

**Keywords:** pear flower, pear leaf, phyllosphere-inhabiting microbes, *Erwinia*, microbiome, fire blight

## Abstract

*Erwinia amylovora* is the causal pathogen of fire blight, a contagious disease that affects apple and pear trees and other members of the family Rosaceae. In this study, we investigated the community dynamics of the pear flower microbiome in an agricultural setting during the naturally occurring infection of *E. amylovora*. Five potential factors were considered: collection date, the flower's phenological stage, location on the tree, location within the orchard, and pear cultivar. The phenological stage and the collection date were identified as the most important factors associated with pear flower microbiome composition, while the location of the tree in the orchard and the flower's location on the tree had a marginal effect. The leaf microbiome reflected that of the abundant phenological stage on each date. The flower microbiome shifted toward *E. amylovora* dominating the community as time and phenological stages progressed, leading to a decreased community diversity. The *E. amylovora* population was represented almost exclusively by six amplicon sequence variants (ASVs) with similar proportions throughout the entire collection period. Other taxa, including *Pseudomonas*, *Pantoea*, *Lactobacillus*, and *Sphingomonas*, were represented by dozens of ASVs, and different succession patterns in their populations were observed. Some of the taxa identified include known antagonists to *E. amylovora*. Overall, our results suggest that flower physiology and the interaction with the environment are strongly associated with the pear flower microbiome and should be considered separately. Taxon-specific succession patterns under *E. amylovora* spread should be considered when choosing candidates for antagonist-based treatments for fire blight.

**IMPORTANCE:**

The spread of pathogens in plants is an important ecological phenomenon and has a significant economic impact on agriculture. Flowers serve as the entry point for *E. amylovora,* but members of the flower microbiome can inhibit or slow down the proliferation and penetration of the pathogen. Knowledge about leaf and flower microbiome response to the naturally occurring spread of *E. amylovora* is still lacking. The current study is the first to describe the Rosaceae flower microbiome dynamics during the naturally occurring infection *of E. amylovora*. Unlike previous studies, the study design enabled us to evaluate the contribution of five important environmental parameters to community composition. We identified different ASV succession patterns across different taxa in the flower consortia throughout the season. These results contribute to our understanding of plant microbial ecology during pathogen spread and can help improve biological treatments for fire blight.

## INTRODUCTION

Microbes inhabit nearly all parts of the plant, from roots to leaves and flowers, both on the plant surface and within it. The phyllosphere microbiome includes microbes that live on leaves, flowers, fruits, and seeds. Microbes inhabiting the phyllosphere contribute to plant health, positively interact with pollinators, and promote plant growth (1–3), but they can also cause several plant diseases (4). Flowers serve as a bacterial entry point to the plant. Their high nutrient content attracts pollinators that serve as carriers or vectors (5) for microbial colonization and succession (6, 7). The flower microbiome can impact the nectar composition (8) and is suspected to play a role in attracting pollinators (9). The phyllosphere microbial composition is affected by plant species, genetics, age, and various environmental conditions such as agricultural management, weather conditions, and soil chemistry (10). The flower microbiome differs even for closely related species such as pear (*Pyrus communis*) and apple (*Malus domestica*). This may be due to the different flower and nectar characteristics, such as pH and sugar content (11). Still, the main factors that shape the phyllosphere microbiome dynamics and its interactions with the plant are not fully understood (6, 10).

The pathogenic, gram-negative enterobacterium *Erwinia amylovora* uses the floral nectar secretory cells as an entry point when infecting Rosaceous fruit trees in fire blight disease (12, 13). Fire blight is a necrotic plant disease affecting apple and pear orchards worldwide, causing severe economic damage (14, 15). Infection results in blights of the shoots, fruit, and rootstock, lowering crop yields and sometimes killing entire orchards (12). Following infection, *E. amylovora* moves through the vascular tissue to colonize the bark and the woody parts of the tree, thus causing blight and creating a bacterial reservoir for future infections (13, 15, 16). The initial *E. amylovora* reservoir in the orchard and its vicinity is affected by infections in the previous years and the quality of the pruning of infected branches in the winter. Weather conditions that favor *E. amylovora* are critical to the disease progression and development. These mainly include warm temperatures coupled with rain or dew during the bloom period (12, 13, 17–20). The current knowledge on microbiome dynamics during the spread of *E. amylovora* in Rosaceous orchards is based on inoculation experiments in which apple flowers were sprayed with *E. amylovora*. These experiments revealed that members of the families *Enterobacteriaceae* and *Pseudomonadaceae* dominate the non-infected flowers, with only small amounts of *E. amylovora* detected. In contrast, the infected flowers were dominated by the inoculated strains (7, 21). Preventive commercial treatments for fire blight are based on antibiotics, copper compounds, and antagonistic bacteria, all with environmental impacts and limited long-term success in the orchard (15, 22–25). Understanding the microbial population dynamics, its relation to *E. amylovora,* and the factors affecting these dynamics might help develop and improve effective, non-destructive methods for preventing fire blight infection.

Here, we aim to explore the effect of different factors on the pear flower microbiome during *E. amylovora* natural infection. We evaluated the contribution of the phenological stage, collection date, location in the orchard, and location on the tree on the microbiome of pear flowers and leaves using 16S small subunit (SSU) rRNA amplicon. Coincidentally, the orchard has undergone an *E. amylovora* infection, which developed into a fire blight event observed later in the season. To our knowledge, this study is the first to document the factors that affect the natural dynamics of the Rosaceous flower microbiome during a naturally occurring infection of *E. amylovora*.

## RESULTS

### Experiment setup

**Fig 1:**
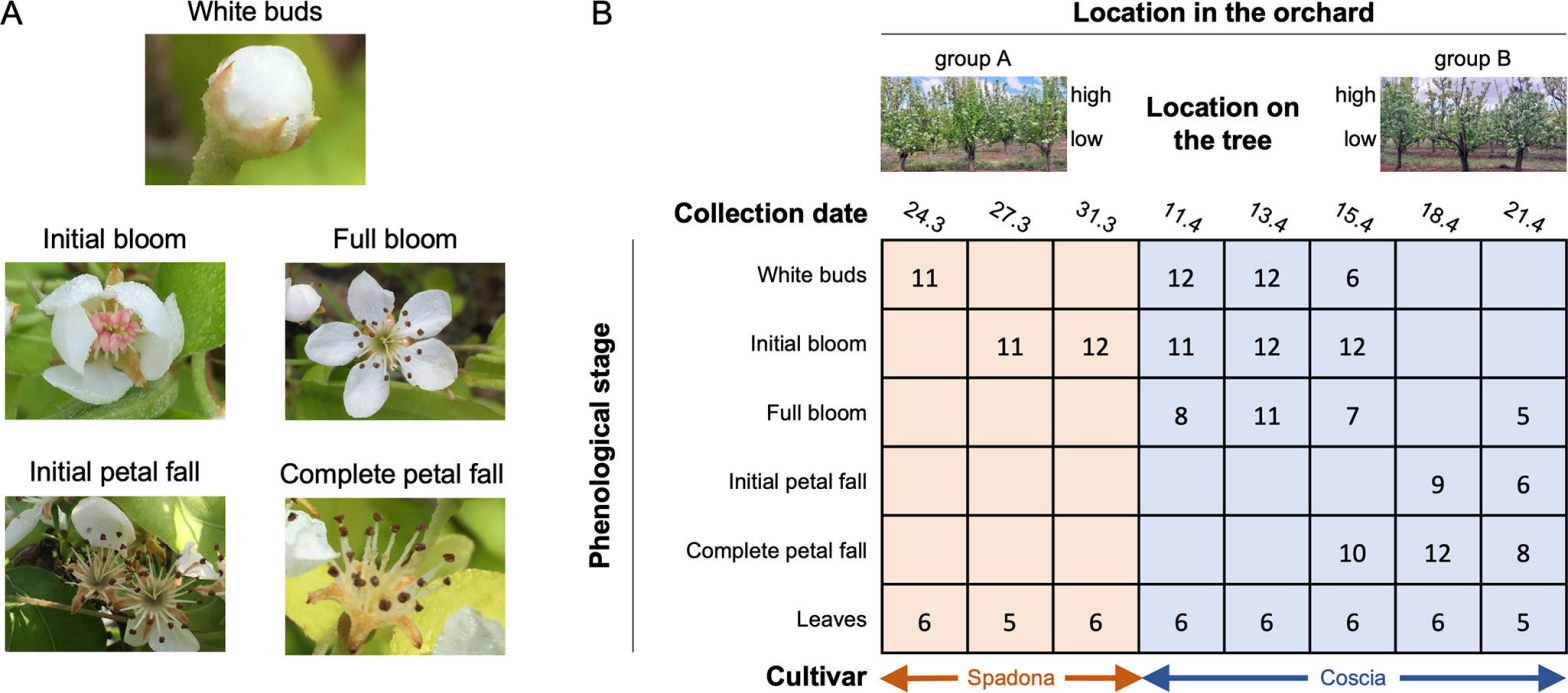
A summary of the collection strategy. (**A**) The flower phenological stages considered in this study. (**B**) Five variables were considered: location in the orchard, location on the tree (high or low), collection date, phenological stage, and cultivar. The numbers indicate the number of samples collected at each collection date/phenological stage. The number of samples used in different analyses may vary according to the rarefaction threshold used.

We aimed to study the factors influencing pear flower microbiome dynamics during an *E. amylovora* infection. To this end, we collected flower and leaf samples during the spring of 2019 from an orchard in the Hula Valley, northern Israel. The study site was selected because it is at high risk for fire blight due to the climatic conditions in the region. Several events of naturally occurring fire blight were documented in the region in the years preceding our study. Five potential determinants were considered: collection date (time), phenological stage, location in the orchard, location on the tree, and cultivar. To investigate the impact of the first four variables, we designed a collection strategy that includes collecting flower and leaf samples from six pear (*Pyrus communis*) Coscia trees on five dates. To assess the impact of the phenological stage on microbial composition, we considered the following five phenological stages: white buds; initial bloom, characterized by open flowers with pink, non-mature anthers; full bloom, in which the anthers matured and opened, and changed color to black; initial petal fall; and complete petal fall (Fig. 1A). For each collection date, two to four abundant phenological stages were selected for sampling. The flower samples were collected from two groups of trees located ~15 m apart, at two heights on each tree (high ≅ 2.5 m; low ≅ 1.5 m), resulting in a total of 12 samples per phenological stage per collection date (Fig. 1B and Materials and Methods). Young leaf samples were collected from each tree on each date, totaling six leaf samples per collection date. To investigate the effect of the cultivar, we collected flowers and leaves from six Spadona trees on three dates earlier in the season, one phenological stage on each date. The Spadona samples were used to compare the microbiome of the white buds and initial bloom stages in the two different cultivars. The samples underwent 16S rRNA amplicon sequencing (V3-V4 region; see Materials and Methods), with an average sequencing depth of ~33K paired-end reads per sample. We identified six abundant *Buchnera* amplicon sequence variants (ASVs) that cover >97% of the *Buchnera* populations, in Spadona flowers only. These ASVs align at 100% similarity to the 16S rRNA gene of the endosymbiont *Buchnera aphidicola*. This suggests that aphids (or their eggs) were present on the flowers. To our knowledge, *Buchnera* is not a part of the flower microbiome, and we, therefore, removed it from our analyses. We collected and sequenced 223 samples (176 flowers and 47 leaves) (Table S1). The samples were rarefied separately for each experiment (see Table S2).

### The effect of the five variables on the microbiome

We identified 29 phyla in the flower and leaf samples of the Spadona and Coscia cultivars (Table S3; Fig. S1). *Pseudomonadota* is the most abundant phylum in both the Spadona and Coscia samples, with an average abundance ranging from 60.7% of the population (11 April [hereafter 11.4], Coscia, initial bloom) to 99.5% (21.4, Coscia, complete petal fall). Other abundant phyla include *Bacillota*, *Bacteroidota*, and *Actinobacteriota*. These phyla were previously reported to be the most abundant in pear flowers, although in different proportions (11). Within *Pseudomonadota*, a significant shift between represented genera is observed when comparing earlier dates and phenological stages (*Pseudomonas*, *Lactobacillus*, *Sphingomonas*, and *Methylobacterium-Methylorubrum*) to later ones, which are dominated by *Erwinia*. These findings are in line with previous studies of Rosaceous trees, in which the families *Enterobacteriaceae* and *Pseudomonadaceae* (phylum *Pseudomonadota*), *Bacillaceae* and *Lactobacillaceae* (phylum *Bacillota*), and the phylum *Candidatus Saccharibacteria* were reported (6, 7, 21, 26). Taxa that occupy nearly all Coscia flower samples and could be considered part of the flower core microbiome (27) could be identified at all taxonomic levels from genus to phylum (Fig. S2). At the genus level, these include *Pseudomonas*, *Sphingomonas, Methylobacterium-Methylorubrum*, and *Hymenobacter*, as well as other genera that were prevalent in most samples. Notably, only four phyla were prevalent in all the samples: *Pseudomonadota*, *Bacillota, Actinobacteriota*, and *Bacteroidota*.

**Fig 2:**
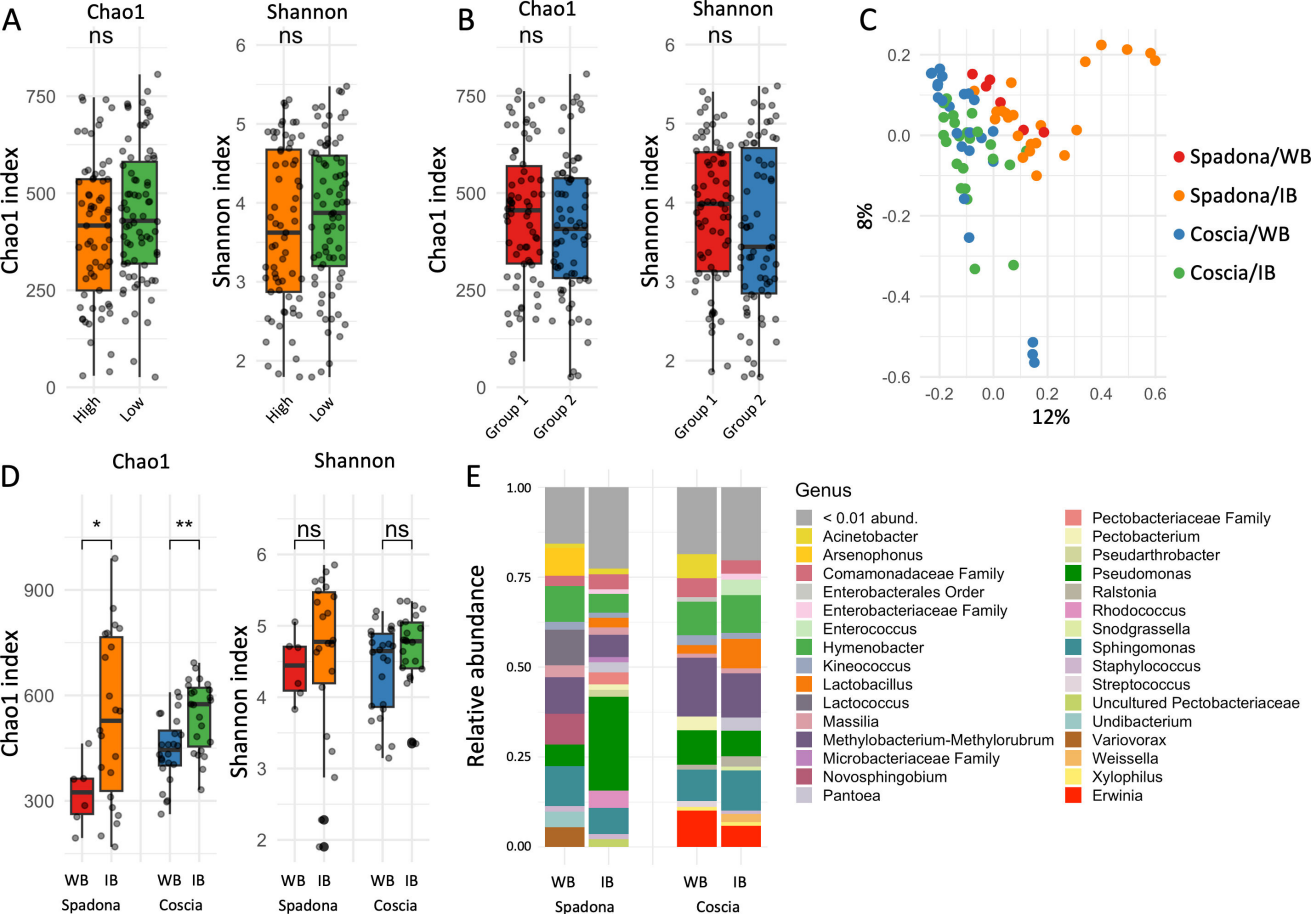
(**A**) Distribution of Chao1 and Shannon indexes for Coscia samples collected at different heights and (**B**) the same for Coscia samples collected at different locations in the orchard. Differences between all pairs are insignificant (Kruskal-Wallis). (**C**) PCoA plot of the white buds (WB) and initial bloom (IB) samples from Spadona (24.3, 27.3, and 31.3) and Coscia (11.4 and 13.4). (**D**) Chao1 (left) and Shannon (right) diversity indexes of the samples, grouped by cultivar and phenological stage. Differences in Shannon diversity between the phenological stages and the cultivars are insignificant (Kruskal-Wallis). The Chao1 index is significantly different (*P*-value = 0.0025, Kruskal-Wallis). (**E**) Relative abundances of genera in the white buds and initial bloom phenological stages, Spadona and Coscia.

**Fig 3:**
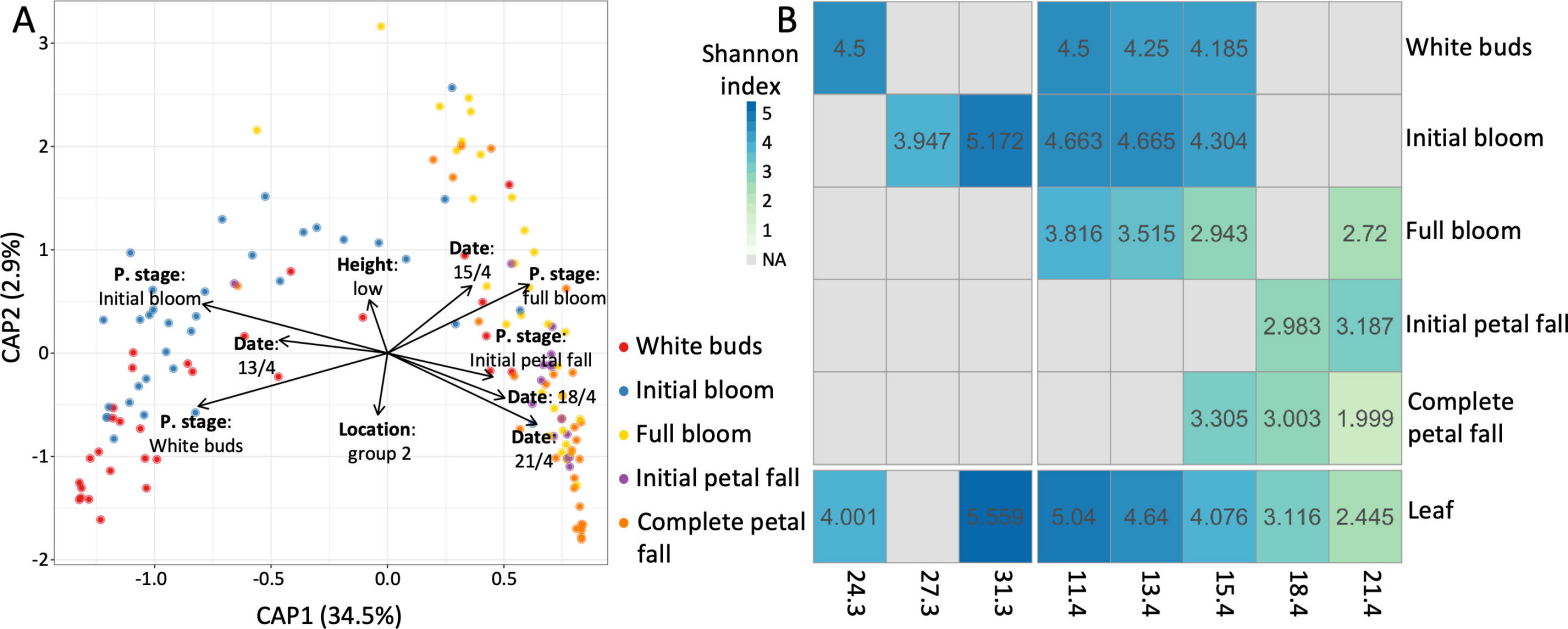
(**A**) Constrained canonical analysis of principal coordinates of Coscia flower samples, using Bray-Curtis dissimilarity. The model applied was phenological stage + collection date + height + location in the orchard. The plot is colored by the phenological stage. (**B**) The mean Shannon diversity over the same dates and phenological stages.

The flower samples collected from different heights in Coscia trees show negligible and marginally significant partitioning when confounding for both the collection date and the phenological stage of the flowers (*R*^2^ = 0.01, *P*-value = 0.005; PERMANOVA; Table S4). The Shannon and Chao1 diversity indexes at different heights are statistically insignificantly different when comparing different heights over the same phenological stages and dates (Kruskal-Wallis; Fig. 2A). The location of the tree in the orchard had a small yet significant effect on both the flower and leaf microbiomes when confounding for both collection date and phenological stage (*R*^2^ = 0.01, *P*-value = 0.002; PERMANOVA; Table S4). Differences in alpha diversity for the location in the orchard were not statistically significant (Kruskal-Wallis; Fig. 2B). In Spadona, the location in the orchard showed a higher yet marginally significant effect on the microbiome (*R*^2^ = 0.1, *P*-value = 0.03; PERMANOVA; Table S4). Constrained canonical analysis of principal coordinates (CAP) of the Coscia flowers shows that the contribution of the height and location in the orchard to the first two dimensions is small, supporting the conclusion that the effect of these variables is small (Fig. 3A). Therefore, we disregarded these variables in the other analyses.

We compared the samples collected from the Spadona cultivar to those from the same phenological stages in Coscia. In this analysis, only samples collected during the first two collection dates of Coscia (11.4 and 13.4) were analyzed due to the spread of *E. amylovora* in the orchard at later dates (see below). Principal coordinates analysis (PCoA) shows that samples from the two cultivars cluster separately regardless of phenological stage (Fig. 2C). In both cultivars, the Chao1 index increased from the white buds to the initial bloom stages (Fig. 2D). Notable differences in relative abundance between Spadona and Coscia include the genera *Erwinia*, *Lactococcus*, *Rhodococcus*, and *Pseudomonas* (Fig. 2E). We note that, since the samples were collected on different dates, it is impossible to determine whether the observed differences are because of the cultivar or the environmental conditions.

**Fig 4:**
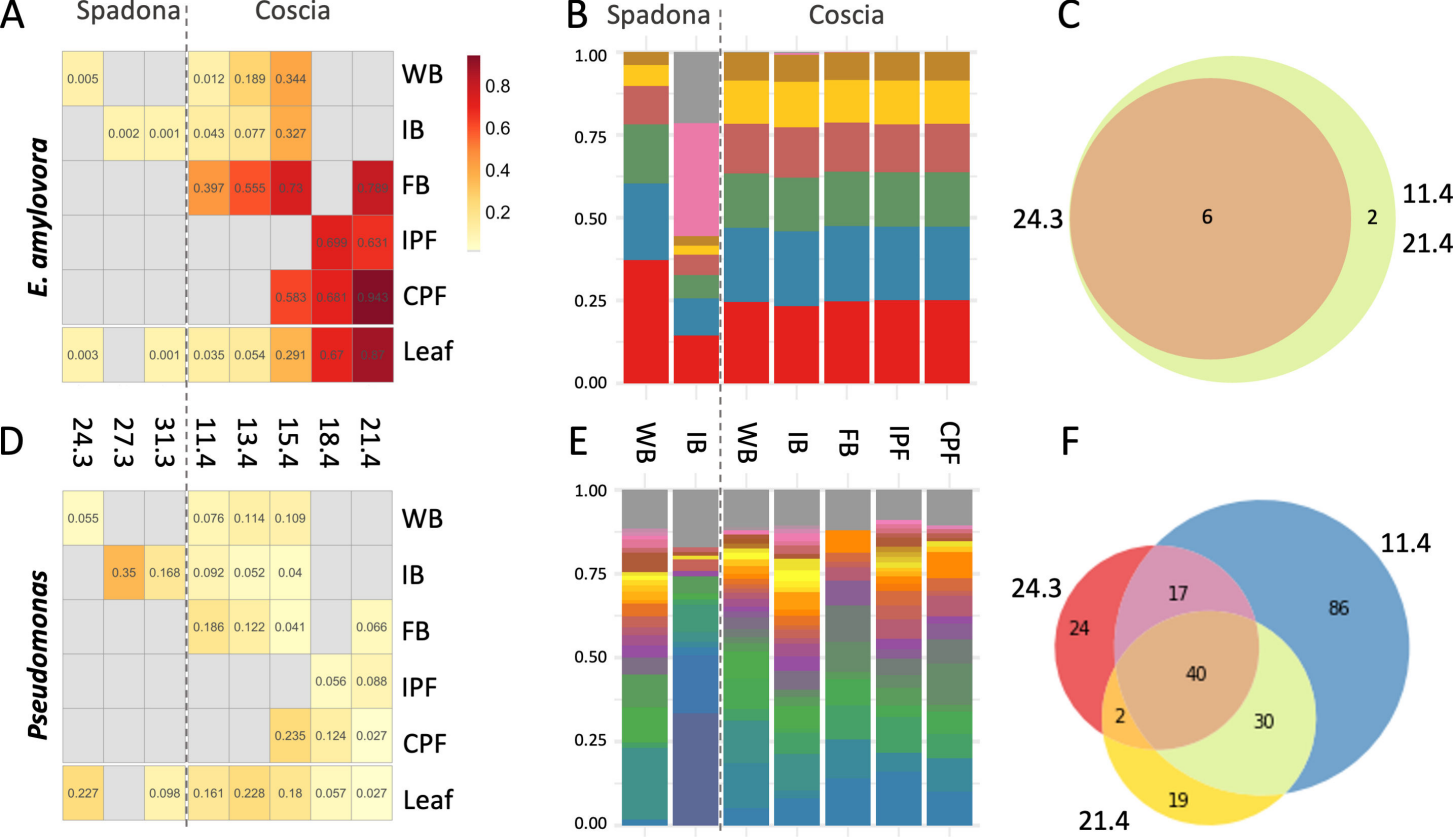
(**A**) The mean relative abundances of ASVs assigned to *E. amylovora* in flowers and leaves across the different collection dates and phenological stages. WB = white buds, IB = initial bloom, FB = full bloom, IPF = initial petal fall, and CPF = complete petal fall. (**B**) The distribution of *E. amylovora* ASVs in each phenological stage. (**C**) The number of *Erwiniaceae* ASVs shared between samples collected on the first collection date for Spadona (24.3), the first collection date for Coscia (11.4), and the last collection date (21.4). (**D through F**) Same as panels A–C for *Pseudomonas*.

We considered the effect of the phenological stage and collection date on Coscia samples through a PERMANOVA model that includes both variables and the interaction term. The collection date (*R*^2^ = 0.25, *P*-value = 0.001; Table S4), the phenological stage (*R*^2^ = 0.17, *P*-value = 0.001; Table S4), and the interaction term (*R*^2^ = 0.06, *P*-value = 0.003; Table S4) all have a significant effect on the microbiome. Community composition demonstrated a shift from the first two to the last three phenological stages (Fig. 3A; Fig. S3A), and from the first two to the last two collection dates, with the middle collection date (15.4) representing a transition between the two groups (Fig. 3A; Fig. S3B). The large effect of the phenological stage and the collection date on the microbiome compared to the location on the tree and in the orchard is also demonstrated by the CAP of the Coscia flowers (Fig. 3A). Community diversity peaked during the first two phenological stages and declined sharply with the spread of *E. amylovora* (Fig. 3B). Significant differences in diversity were observed among samples collected on different dates during the full bloom and complete petal fall stages, as well as for leaves, but no significant differences were found for the other phenological stages (Kruskal-Wallis; Table S5). Diversity also varied significantly across phenological stages on all dates except 18.4 (Kruskal-Wallis; Table S6). The leaf microbiome diversity changed significantly over time and was overall similar to the one of the abundant phenological stage at each date (Fig. 3B). These differences are mostly due to the spread of *E. amylovora* (discussed later). A breakdown of the samples to phenological stages at each date, and to collection dates at each phenological stage, shows distinct clustering of groups that are dominated by *E. amylovora* and those that are not (Fig. S4 to S6). These groups also differ in community diversity (Fig. 3B and 4A), demonstrating the influence of both phenological stage and collection date on the microbiome composition.

### Estimating pear flower bacterial population sizes over different phenological stages

**Fig 5:**
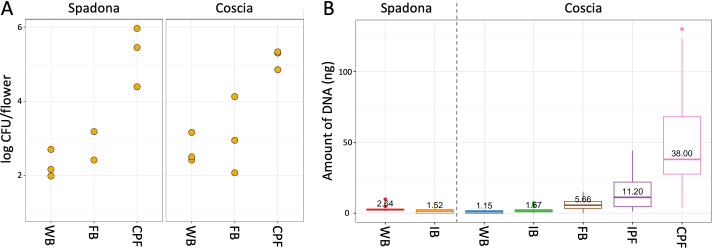
(**A**) Colony forming unit counts for Coscia and Spadona flowers of varying phenological stages. (**B**) Amount of DNA (ng) extracted from the study samples, as estimated by a fluorometer.

We conducted colony forming unit (CFU) count assays to estimate the bacterial population sizes on the flowers in three phenological stages: white buds, full bloom, and complete petal fall. Flowers from these three stages were collected during two dates in April 2024 from an orchard located 17 km south of the original orchard. Bacteria were grown on nutrient agar (NA) plates. Overall, CFU counts increased from 10^2^–10^3^ at the white buds stage to 10^2^–10^4^ at the full bloom stage, to approximately 10^5^–10^6^ at the complete petal fall stage (Fig. 5A). These numbers are consistent with those observed previously in apple flowers (28). The proportion of *E. amylovora* in the communities evaluated in this experiment is unknown, but fire blight was observed later in the season in the orchard.

We also considered the DNA yield of the original (2019) samples as a proxy for the population size. DNA yield is directly related to the number of cells and was previously used as an inaccurate estimator of population sizes (29). In the 2019 samples, the DNA yield significantly increased with the advance of the phenological stage, while the proportion of mitochondria and chloroplasts decreased, indicating a decrease in the overall proportion of plant DNA in the samples (Fig. 5B; Fig. S7). DNA yield alone was insufficient for accurate population size estimations; still, its overall trend fits the one observed in the CFU assays.

### The spread of *Erwinia amylovora* in the orchard

The orchard sampled has a history of fire blight caused by natural infections and inoculation experiments performed in previous years. During the collection season, optimal conditions for the spread and proliferation of *E. amylovora* occurred several times (Fig. S8). With the progression of the date and the phenological stage, the flower and leaf microbiomes became dominated by members of the family *Erwiniaceae*.

To evaluate the large *Erwiniaceae* populations in our samples, we further analyzed all *Erwiniaceae* ASVs in our data by comparing them to the core_nt database of NCBI. We identified six ASVs that dominated the *Erwiniaceae* population aligning at 99.8–100% to the 16S rRNA genes of the type-material *E. amylovora* ATCC 15580 (Fig. S9A). The second-best type-material hit with a complete genome was *Erwinia pyrifoliae* DSM 12163, another blight-causing pathogen in pears (30). Other *Erwinia* type-material strains with genomes aligned at over 98% similarity were all confirmed to be *E. amylovora* strains (Fig. S9B). Two other ASVs aligned at 99.35% and 99.75% to the 16S rRNA gene of *E. amylovora*. These two ASVs appeared in two and three copies, compared to over 161K copies of each of the six abundant ASVs. All other *Erwiniaceae* ASVs aligned to other *Erwiniaceae* species.

In Coscia flowers, the relative abundance of *E. amylovora* ASVs increased significantly with the progression of both the collection date and the phenological stage until reaching ~95% of the community in the last date and phenological stage. The relative abundance of *E. amylovora* on leaves increased steadily to >85% in the last collection date (Fig. 4A). The relative abundance of most lineages declined over time as *E. amylovora* spread, with a few exceptions such as *Rosenbergiella* at the initial petal fall stage (Fig. 4D; Fig. S4).

The six ASVs aligned with the 16S rRNA gene of *E. amylovora* at 100% similarity were identified in all collection dates of the Spadona and Coscia flowers (Fig. 4B and C). The genome of *E. amylovora* includes seven copies of the 16S rRNA gene that differ by a few nucleotides; therefore, it is impossible to determine whether the ASVs represent one or multiple strains. Other lineages, such as *Pseudomonas*, *Pantoea*, *Lactobacillus*, and *Sphingomonas*, were represented by dozens of ASVs. Some ASVs were detected only at specific time points, while others were present throughout the entire collection period, appearing from the first to the last sampling dates (Fig. 4D through F; Fig. S10 and S11B). These trends suggest that succession had occurred during the collection period, possibly as a response to the spread of *E. amylovora*. The alpha diversity of *Pseudomonas* populations in each sample did not change significantly throughout the collection period despite their decline in relative abundance. On the other hand, the diversity of *Sphingomonas* declined, and the diversity of *Lactobacillus* demonstrated mixed trends (Fig. S10 and S11).

## DISCUSSION

In this study, we describe the pear flower microbiome during the infection of *E. amylovora*. The weather conditions in the orchard during the study period favored fire blight disease, and disease signs were observed later in the flowering period. To date, only a few amplicon-based studies targeted the pear flower microbiome, with most studies on the Rosaceous flower microbiome focusing on apple trees. The prevalent phyla found in this study were previously reported in apple and pear microbiomes (6, 21, 26). The effect of the *E. amylovora* population on the leaf and flower microbiomes and the dynamics of the plant microbiome during the infection of *E. amylovora* were mostly studied through culturing and inoculation experiments. To our knowledge, the current study is the first to describe microbial community dynamics during a naturally occurring infection of *E. amylovora*.

We aimed to reveal the contribution of five factors to the pear flower microbiome dynamics: the height of the flowers on the tree, the location of the tree in the orchard, the phenological stage, the collection date, and the cultivar. Our results demonstrate small yet significant differences in the flower microbiome between different heights despite a relatively small (~1 meter) difference in the heights compared. These differences may be due to continuous differences in the flowers' exposure to environmental conditions such as sun hours and wind that can directly affect temperature and humidity. The microbiome of trees at different locations in the orchard was overall similar but exhibited small yet significant differences. We attribute these differences to local environmental factors and microbial community processes that affect different parts of the orchard and are noticeable even over distances of 10–20 meters.

Our results show that Spadona and Coscia cultivars have slightly different microbiomes during the first two phenological stages (white buds and initial bloom). The Spadona trees bloomed 2–3 weeks before the Coscia trees during the 2019 flowering season studied here. Therefore, the differences are potentially due to different environmental conditions. In apples, the cultivar did not significantly affect the microbial composition when compared during the same period (26).

The phenological stage and the collection date are interrelated but represent different factors. The phenological stage represents the flower physiology (e.g., the exposure of the flower stigma), nectar availability and composition, and flower age. The collection date is related to external factors, in particular, weather and exposure to pollinators. Our experiment was heavily influenced by the spread of *E. amylovora* in the orchard, which became the most abundant bacterial species in the flower and leaf communities. Still, flowers collected at different phenological stages over the same dates exhibited distinct community structures, as did flowers in the same phenological stage collected over different dates. These differences were significant and highlight the need to consider both factors when designing experiments aimed at studying the flower microbiome. Interestingly, the results show that leaf microbial populations reflect the dominant phenological stages on each date.

The factors considered in this study explain roughly half of the variance in the observed community structure. Other factors may include the weather (rain, temperatures, and exposure to sun and wind), the turn of the tree, and pollinators in the orchard. The turn of the tree could affect the flower microbiome as different turns are exposed to different conditions. For example, east-facing flowers could dry up faster than flowers facing other directions in the morning. Pollinators, observed and active in the orchard during the collection period, are known contributors to microbial dynamics (5). Their preferences and behavior may also contribute to differences across locations in the orchard and height (5, 31), but these were not considered in the current study. Microbial features such as phages (32) or strain and species heterogeneity may also affect community compositions in ways that are difficult to model using this study's methods.

The flowering season in the late spring has climatic conditions favored by *E. amylovora*. The 2019 flowering season had 14 days (out of 45) that met the criteria of the “Fire Blight Control Advisory” decision support system for fire blight high-risk weather (33) (Fig. S8). The pathogen, which was present at ~1% of the population in Coscia white buds at the beginning of the season, occupied nearly 95% of the population in the last collection date and phenological stage. The prevalence of *E. amylovora* differed across phenological stages and collection dates, in line with previous studies (17, 33). Community diversity increased from the white buds to the initial bloom stages before the spread of *E. amylovora* but then declined until the last phenological stage and collection date following the spread of *E. amylovora*. These dynamics are expected and are different from those observed in healthy bacterial communities of apples and pears (6, 11). The same six *E. amylovora* ASVs were observed in similar proportions throughout the entire collection period and accounted for nearly 99.8% of the *Erwinia* ASVs identified.

In contrast to the microbiome diversity, which declined with time, the species and strain diversity of the different taxa varied. The genus *Pseudomonas*, for example, maintained a similar diversity over the collection period, even when its relative abundance in the population decreased. The diversity of *Sphingomonas*, on the other hand, declined with time. Previous studies showed that *Pseudomonadaceae* was negatively correlated with *E. amylovora* when testing the microbiome of single flowers (7, 21). In our study, samples consist of 30 flowers, thus representing an average of multiple blossoms. Therefore, it is not possible to determine whether *E. amylovora* and the other taxa are evenly spread over the different flowers or if the communities of specific flowers differ greatly. We estimate that the number of bacterial cells on the flowers increased by several orders of magnitude from the beginning to the end of the flowering season. The share of *E. amylovora* in the community increased by two orders of magnitude from the beginning to the end of the season. The number of cells in the CFU assay, which was done on samples collected in 2024, increased by two to four orders of magnitude. While the exact share of *E. amylovora* cells in the CFU assay is unknown, this suggests that, even if *E. amylovora* spread in the orchard in 2024, the number of non-*Erwinia* cells may have increased over time and phenological stages despite its shrinking relative abundance.

Biocontrol by antagonistic microbes has been proposed as a method for developing new treatments against fire blight. While the discovery of candidate antagonists is beyond the scope of this work, our results suggest that bacterial populations overall maintain their diversity in the presence of *E. amylovora,* with ASV turnover occurring throughout the season. Assuming that an antagonistic bacterium native to the pear flower microbiome is found, our results suggest that it may face competition from close species and strains when introduced to the orchard. Therefore, we anticipate that introducing antagonists alone would achieve limited success unless the microbiome is manipulated (e.g., using phages) or the introduced strain is engineered to include functions allowing it to compete with the other close species and strains. Searching for antagonists from one of the taxonomic groups identified as the core microbiome may increase the chances of success of this approach.

### Conclusion

In our experiment, the share of *E. amylovora* in the community increased from less than 1% of the flower microbiome on the first collection date to more than 95% on the last date and phenological stage. Other groups, such as *Pseudomonas*, *Sphingomonas*, and *Lactobacillus*, declined in overall abundance but were represented by dozens of ASVs with significant turnover throughout the season. The collection date and the phenological stage both explain a significant portion of the microbiome population composition during the spread of *E. amylovora*, while the cultivar, the location in the orchard, and the location on the tree have a small effect on the flower microbiome.

## MATERIALS AND METHODS

### Collection site and weather

Samples were collected from 12 pear trees (*Pyrus communis* cultivars Spadona and Coscia) at an orchard in the Hula Valley in northern Israel (33.1505N, 35.6261E) during March and April 2019. The trees were 10 years old. The plot has a history of fire blight, and inoculation experiments with *E. amylovora* were performed in previous years. The plot was treated with pesticides using the conventional local protocol in previous years but not in 2019. The undergrowth was sprayed with herbicides before the flowering period. Weather data (Fig. S1) were collected by a meteorological station adjacent to the orchard. The station provides meteorological data to alert farmers of fire blight favorable conditions based on the Fire Blight Control Advisory (FBCA) system (33). *E. amylovora* infection alerts were reported by the FBCA system multiple times during the flowering period.

### Sample collection strategy

The sample collection strategy and the number of samples that were collected are summarized in Fig. 1. For each cultivar, two groups of three neighboring trees were sampled (a total of six trees for each cultivar) to evaluate the variation in microbiome composition resulting from location within the orchard. The groups were located ~15 meters apart. To test the variation in microbiome composition resulting from height, we collected flowers at heights ~1.5 and ~2.5 meters (Fig. 1A). Spadona flowers and leaves were collected on three dates between 24.3 and 31.3, and Coscia samples were collected on five dates between 11.4 and 21.4. All samples were collected during the morning hours. For the Coscia cultivar, flowers were sampled from two to four prevalent phenological stages in each collection date. For the Spadona cultivar, flowers of a single phenological stage were collected on each date (Fig. 1B and C). Overall, 12 samples were collected for each phenological stage at each date. One leaf sample was collected at height ~1.5 meters from around each tree at all collection dates (six samples per collection date).

### Sample collection

We collected 30 flowers for each flower sample, following reference 6, and 15 leaves were collected for each leaf sample. All flowers and leaves were collected using sterilized tweezers, and samples were immediately placed in liquid nitrogen, transported to the laboratory, and stored in −80°C until processing.

### Bacterial cell number estimations

The flowers for the bacteria count data were collected on 8 April 2024 (Spadona) and 24 April 2024 (Coscia). The flowers were collected from an orchard located 17 km south of the original orchard (32.9968N, 35.5843E) as described above and were transferred on ice to the laboratory. For each phenological stage, a pool of 15 flowers was homogenized in PBS, diluted, plated on Nutrient Agar (made from Difco Nutrient Broth), and incubated at 28°C for three days before counting.

### DNA extraction and amplification

We applied the DNA extraction protocol described in reference 6 with minor modifications. The samples were processed a few weeks after collection. The extraction process included steps to separate the microbial cells from the plant material and to reduce the amount of chloroplasts and mitochondria. Samples were taken from the freezer and thawed on ice for ~30 minutes. Next, we added 35 mL 1× PBS–0.15% Tween solution, with 5–7 glass beads as a mild abrasive. The samples were then put into an orbital shaker at 270 rpm for 20 minutes at 4°C, followed by a 5 minute sonication in a water bath (Elmasonics S30H sonicator). The flower debris was removed using a sterile gauze cloth into a new, sterile tube. The samples were centrifuged at 272 g for 5 minutes (Eppendorf 5810R) at 4°C, to remove all remaining flower debris. The top liquid was centrifuged at 5,111 g for 30 minutes at 4°C. The pellet was collected, and the DNA was extracted using the Exgene Cell DNA Isolation kit sv100 prep, following the manufacturer's instructions. DNA concentrations were measured using the Invitrogen Qubit Fluorometer. Four blank samples were prepared and sequenced, starting at the separations stage, and all resulted in a low yield of reads (a few hundred). None of the ASVs detected in these samples were detected in significant quantities in other samples.

Sequence libraries were prepared and sequenced at the Research Resources Center of the University of Illinois in Chicago. Libraries were prepared using the Nextera XT DNA Library Preparation kit and sequenced on Illumina MiSeq sequencer (2 × 250 bp). The v3-v4 region of the 16S ribosomal small subunit was amplified using the 341 f/785 r primer set 341 f (5′-CCTACGGGNGGCWGCAG-3′), 785 r (5′-GACTACHVGGGTATCTAATC-3′). These primers were previously found to recover a high diversity of plant-associated bacteria (34). Samples with low sequence counts or high chloroplast contamination were dropped from the analysis. Overall, 176 flower samples (34 Spadona and 142 Coscia) and 47 leaf samples (17 Spadona and 30 Coscia) were analyzed (Fig. 1B; Table S1). Four control samples were prepared by the sequencing center in addition to the ones we provided, and all resulted in only a few hundred reads. The ASVs detected in these samples were not observed in other samples.

### Sequence analysis and statistics

All the initial analyses were performed using qiime2 v2022.8 (35). We processed the paired-end reads using the Dada2 plugin using the “pseudo” option (36). We extracted 18,507 unique amplicon sequence variants (ASVs; also termed features) from 7,500,628 sequences that passed quality control, an average of 33,635 reads per sample.

Amplicon sequence variants were aligned using mafft (37), and phylogeny was constructed using fasttree (38). The taxonomy was assigned using the q2-feature-classifier plugin with the classify-sklearn option (39) against release 138 of the SILVA database (40), SSURef NR99 collection. The SILVA taxonomy was manually modified to achieve consistency with the latest nomenclature: *Proteobacteria* was renamed *Pseudomonadota*, *Firmicutes* was renamed *Bacillota*, and *Cyanobacteria* was renamed *Cyanobacteriota*. Mitochondria and chloroplast sequences were filtered out. Some samples contained ASVs assigned to the *Buchnera* genus with 100% identity to *B. aphidicola*, an endosymbiont of aphids. These ASVs were removed from subsequent analysis. We also removed ASVs present at less than 0.0001% of the reads. Overall, 18,451 ASVs were retained. The samples were rarefied (41) separately for each analysis using phyloseq. Refer to Table S2 for exact numbers.

The downstream analyses, graphs, and statistics were done in R, using the phyloseq (version 1.36.0) (42) and the vegan (43) packages. Alpha diversity was computed through the Shannon index using phyloseq. Beta diversity was calculated using the vegan package. Constrained ordination was calculated with the ordinate function in phyloseq, using “CAP” method and Bray-Curtis distances. PERMANOVA (permutational multivariate analysis of variance) analysis (44) was computed using the adonis function in vegan (version 2.5.7) with 9,999 permutations. Comparisons of alpha diversities were done using the Kruskal-Wallis tests. To identify their origin, we aligned all *Erwiniaceae* ASVs against the NCBI nt database using BLAST (45). We used the plot_core() function of the R package microbiome for the core microbiome analysis with parameter min_prevalence set to 0.2.

## Data Availability

Raw sequencing data for the project are available via SRA with BioProject identifier PRJNA1043619. The code used for the analyses in this paper is available from https://github.com/SharonLab/pear-microbiome.

## SUPPLEMENTAL MATERIAL

Supplemental figures msphere.00011-25-s0001.pdf Figures S1 to S11.

Supplemental tables msphere.00011-25-s0002.xlsx Tables S1 to S6.

ASM does not own the copyrights to Supplemental Material that may be linked to, or accessed through, an article. The authors have granted ASM a non-exclusive, world-wide license to publish the Supplemental Material files. Please contact the corresponding author directly for reuse.
