# Supplementary material for: Pear flower and leaf microbiome dynamics during the naturally occurring spread of Erwinia amylovora
Source: mSphere. 2025 May 5;10(5):e00011-25. doi: 10.1128/msphere.00011-25 (PMC12108086; doi:10.1128/msphere.00011-25)
Supplement: Supplemental figures — Figures S1 to S11. [file msphere.00011-25-s0001.pdf]

Supplementary Information, pear flower and leaf microbiome dynamics during the naturally occurring spread of *Erwinia amylovora*

Aia Oz[1,*], Orly Mairesse[2,3,*], Shira Raikin[2], Hila Hanani[1], Hadar Mor[1], Mery Dafny Yelin[2,#] and Itai Sharon[1,3,#]

[1] Migal – Galilee Technology Center, Kiryat Shmona, Israel
[2] Northern Agriculture Research & Development, Migal–Galilee Technology Center, Kiryat Shemona, Israel
[3] Faculty of Sciences and Technology, Tel-Hai Academic College, Upper Galilee, Israel
[#] Corresponding authors, itaish@migal.org.il, merydy@migal.org.il
* These authors contributed equally to this work

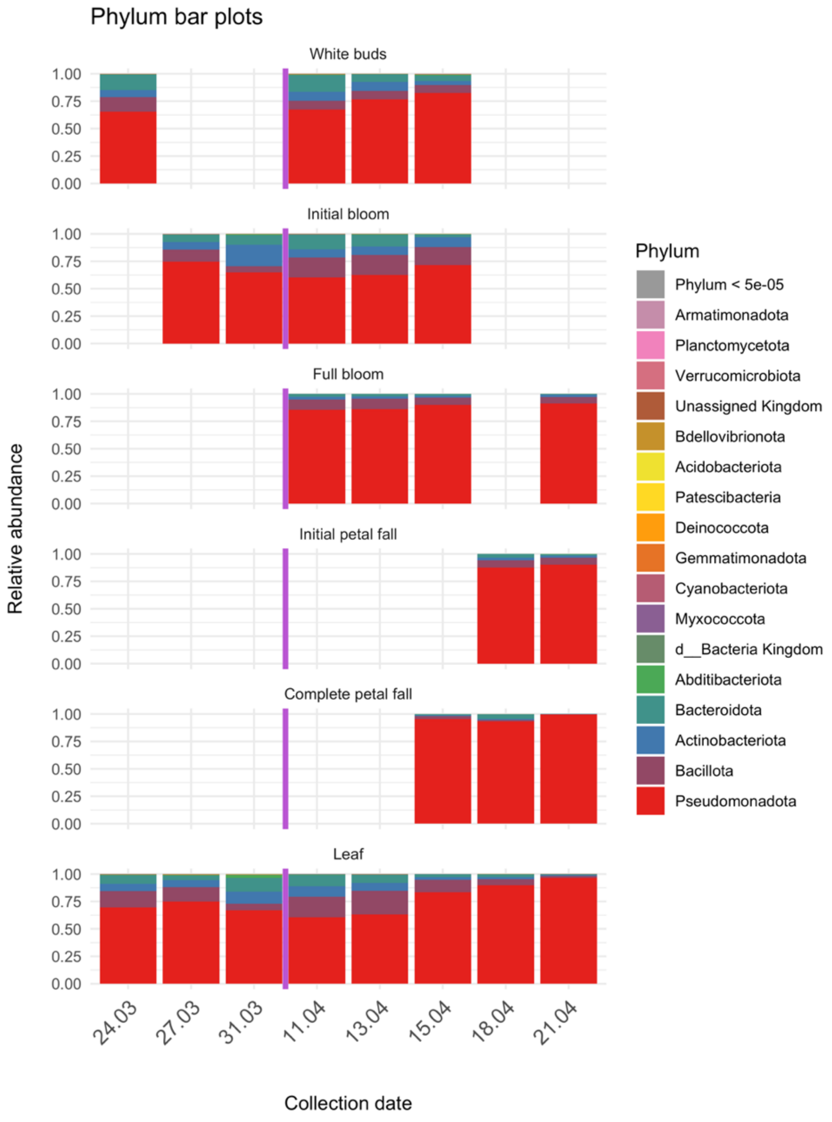

Figure S1: Breakdown of community composition at the phylum level by collection date and phenological stage. The purple lines separate Spadona collection dates (24.3, 27.3, and 31.3) from Coscia collection dates (the rest).

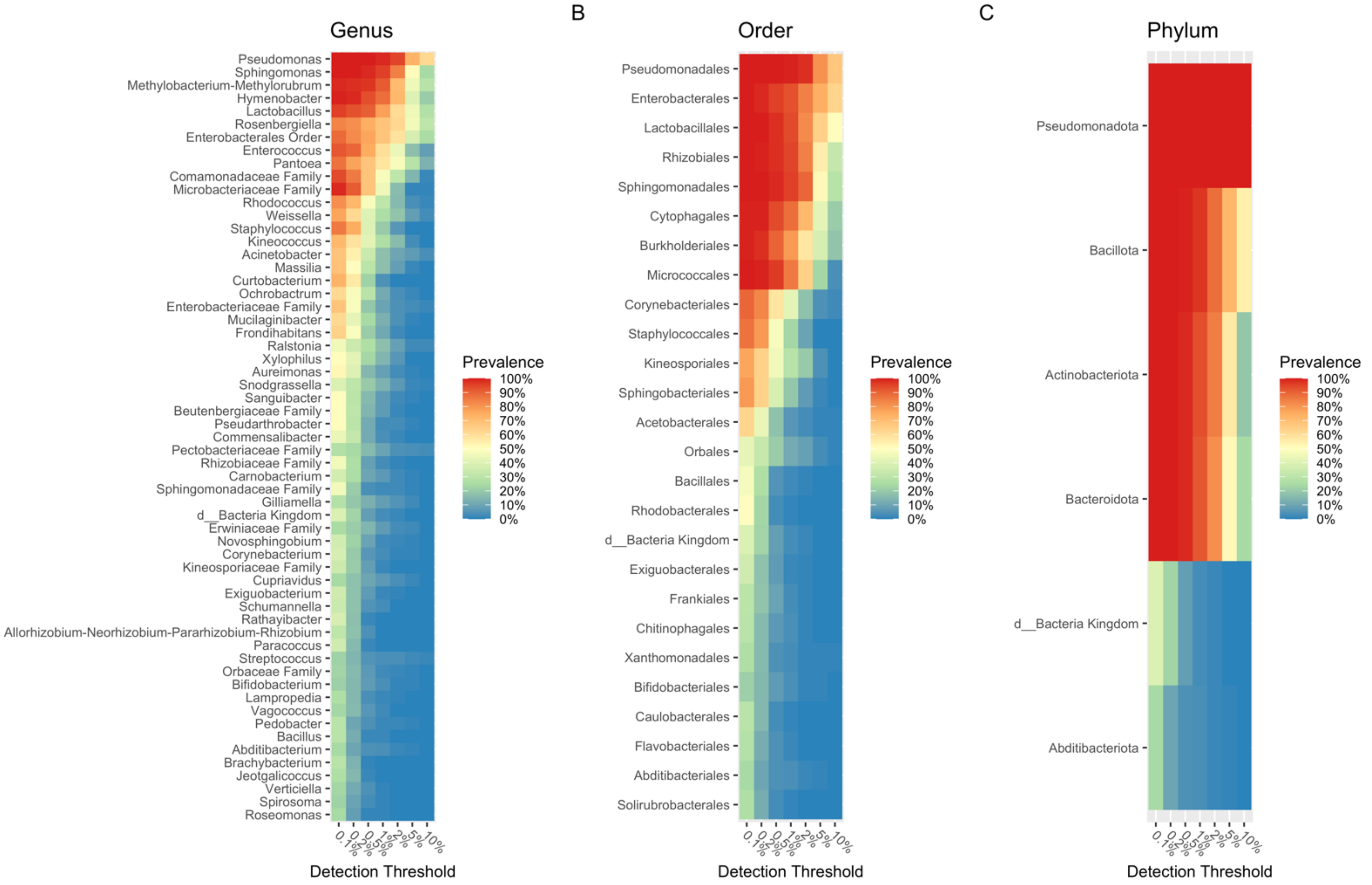

Figure S2: Core microbiome analyses at the genus (A), order (B), and phylum (C) levels for Coscia flower samples of all phenological stages. Colors represent the prevalence in the samples for each taxon (Y-axis) when considering detection thresholds from 0.1% to 10% (X-axis). Only taxa present in at least 20% of the samples are reported. *Erwinia amylovora* was excluded from this analysis.

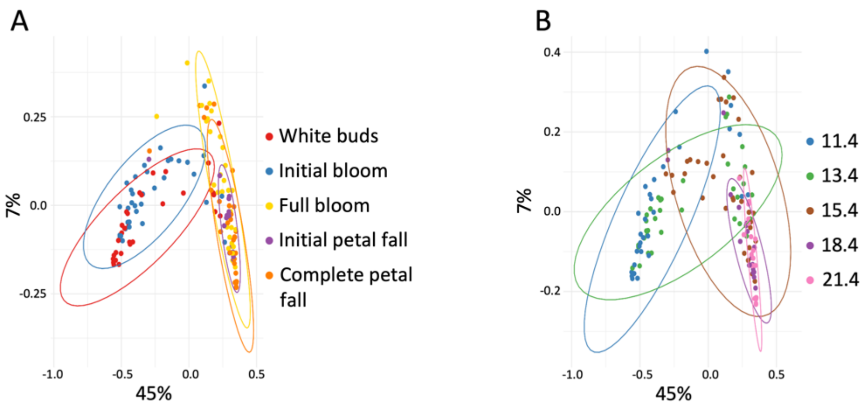

Figure S3: (A, B) PCoA (Bray-Curtis) of Coscia flower samples colored and grouped by phenological stage (A) and collection date (B)

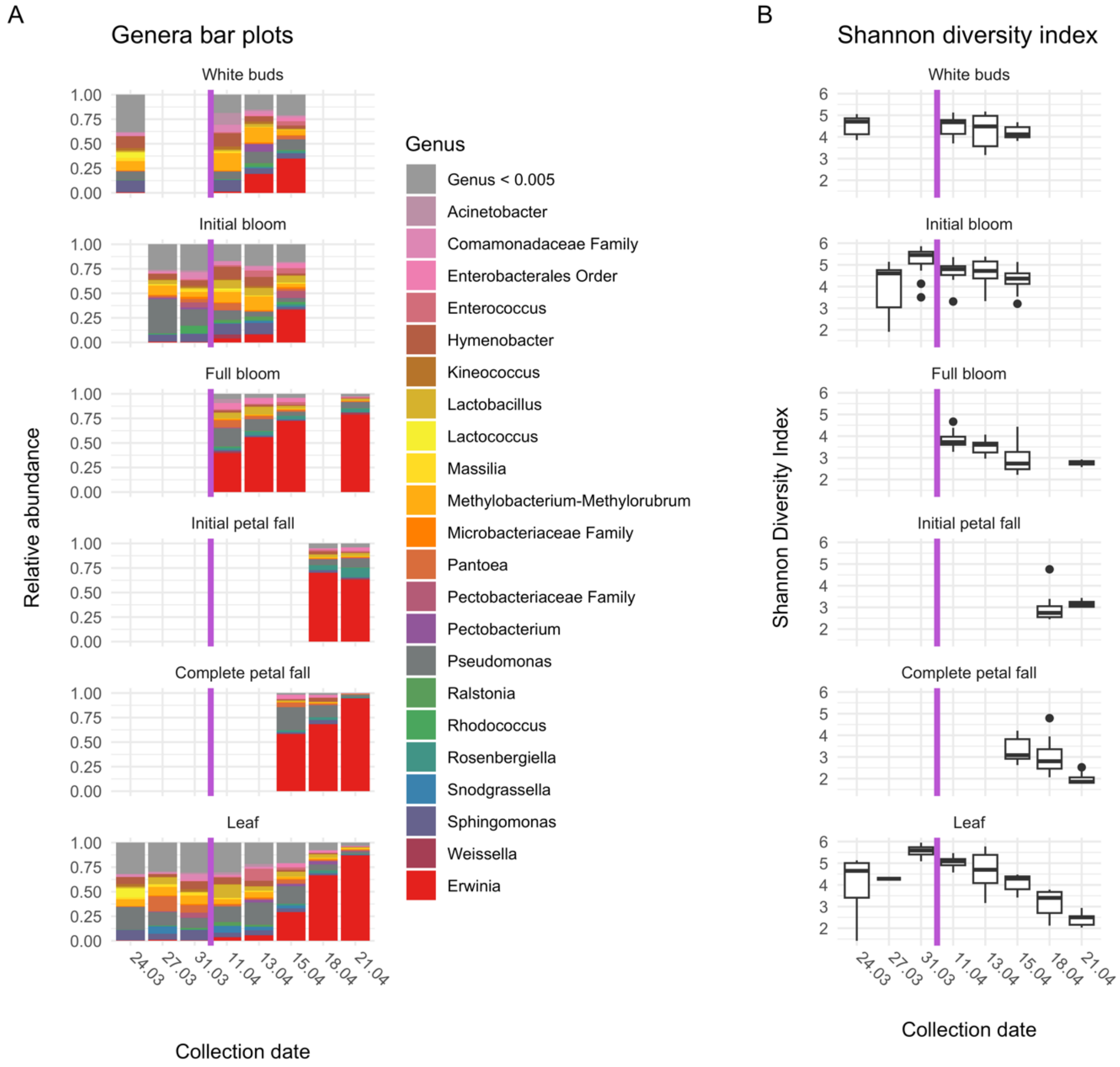

Figure S4: (A) The most abundant genera throughout the collection dates and phenological stages. The purple lines separate Spadona collection dates (24.3, 27.3, and 31.3) from Coscia collection dates (the rest). (B) Shannon diversities at different collection dates and phenological stages. Shannon diversities are significantly different among the following Coscia phenological stages: full bloom (adjusted P-value=0.007), Complete petal fall (adjusted P-value =0.001), and leaves (adjusted P-value=0.001). The Shannon diversity also differed among the phenological stages of the following dates: 11.4 (adjusted P-value =0.002), 13.4 (adjusted P-value=0.002), 15.4 (adjusted P-value=0.002), and 21.4 (adjusted P-value=0.001). All P-values are calculated using the Kruskal-Wallis rank sum test and corrected using FDR.

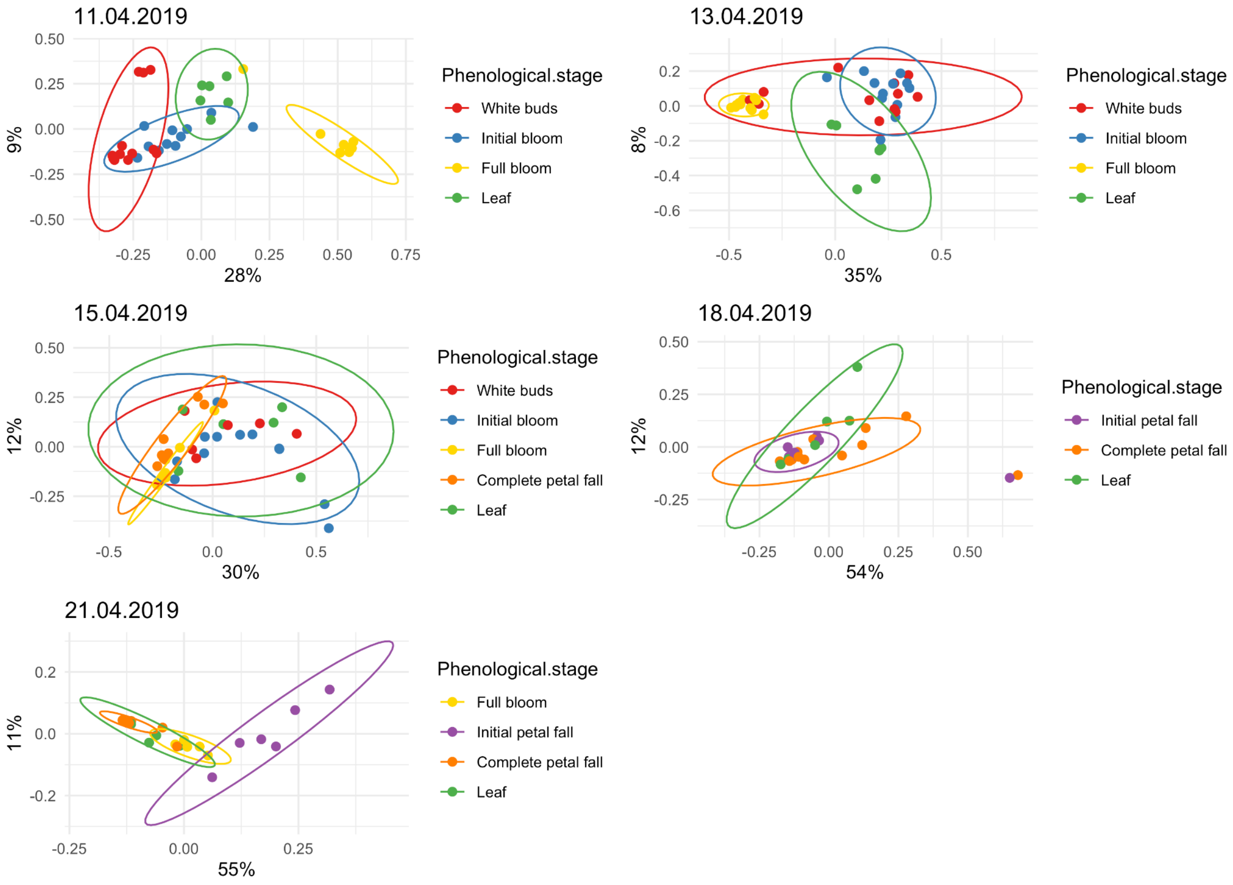

Figure S5: PCoA of the different phenological stages at each collection date (Coscia)

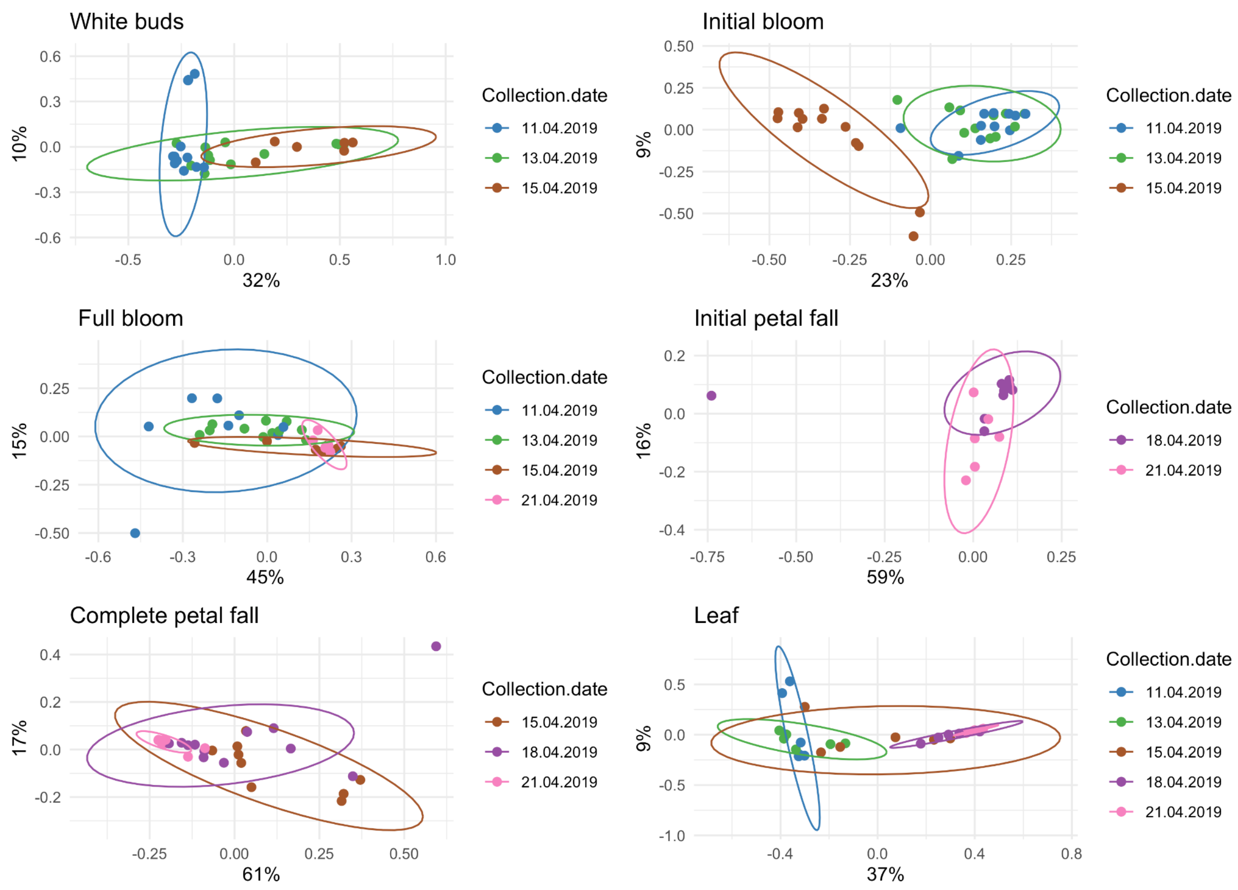

Figure S6: PCoA of the different collection dates for each phenological stage (Coscia)

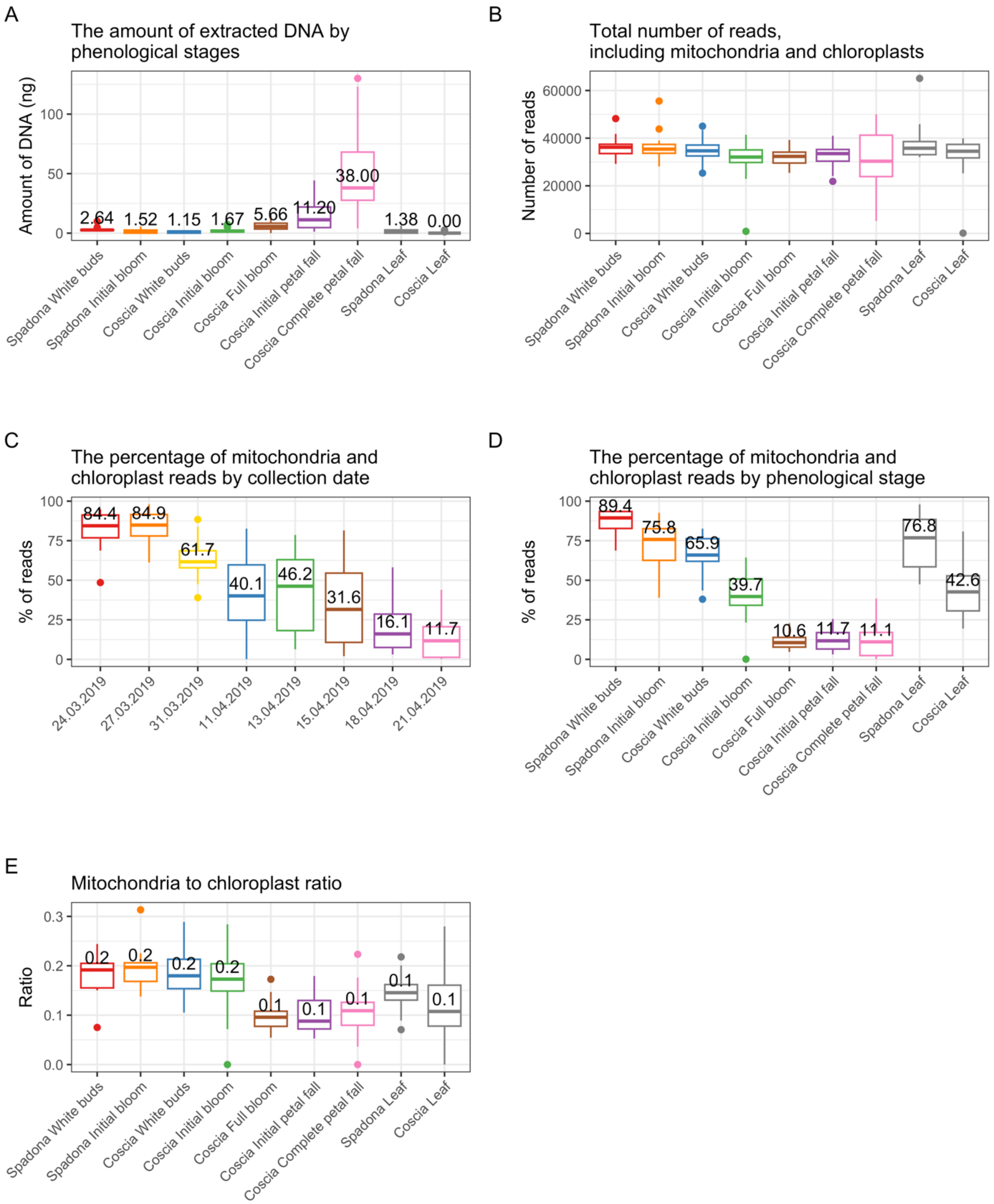

Figure S7: DNA yields as a proxy for calculating the bacteria number. (A) The amount of extracted DNA by phenological stage. (B) The total number of reads sequenced for each phenological stage. The median number is relatively constant at ~35K reads. (C) The percentage of plant mitochondria and chloroplast reads out of the total reads grouped by collection date. (D) Similar to (C) by cultivar and phenological stage. (E) The ratio of mitochondria to chloroplast reads.

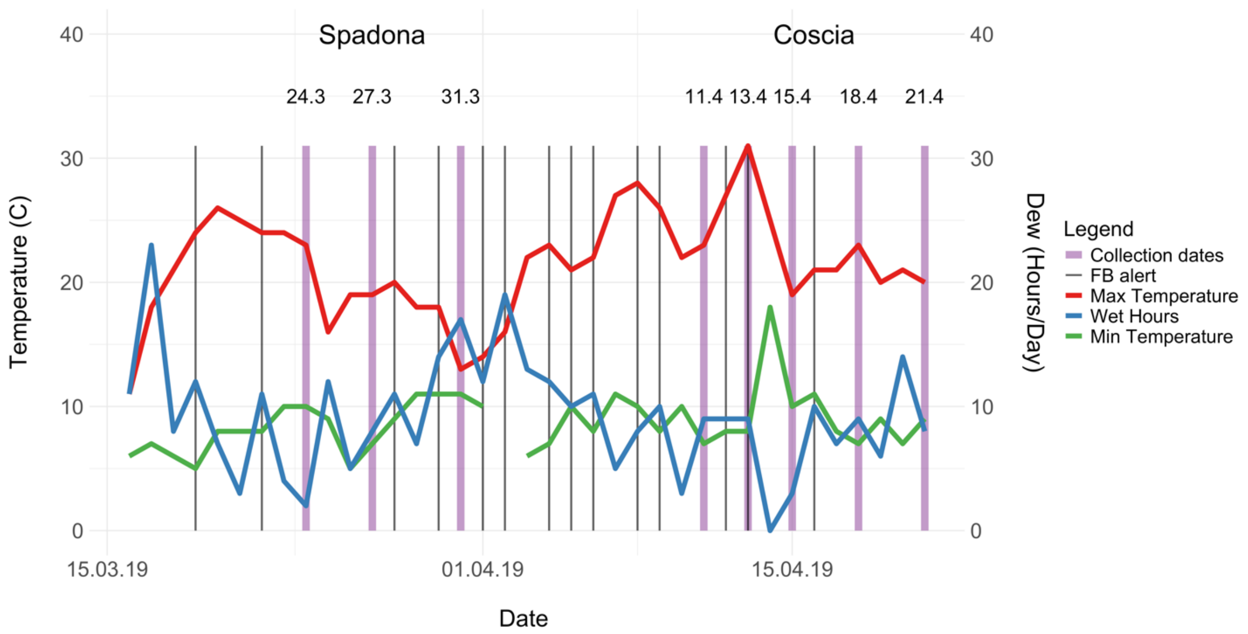

Figure S8: weather conditions during the collection period. Red: maximum temperature; green: minimum temperature; blue: number of wet hours. Black lines show dates in which a fire blight alert was issued by the FBCA system. Purple lines mark the eight collection dates.

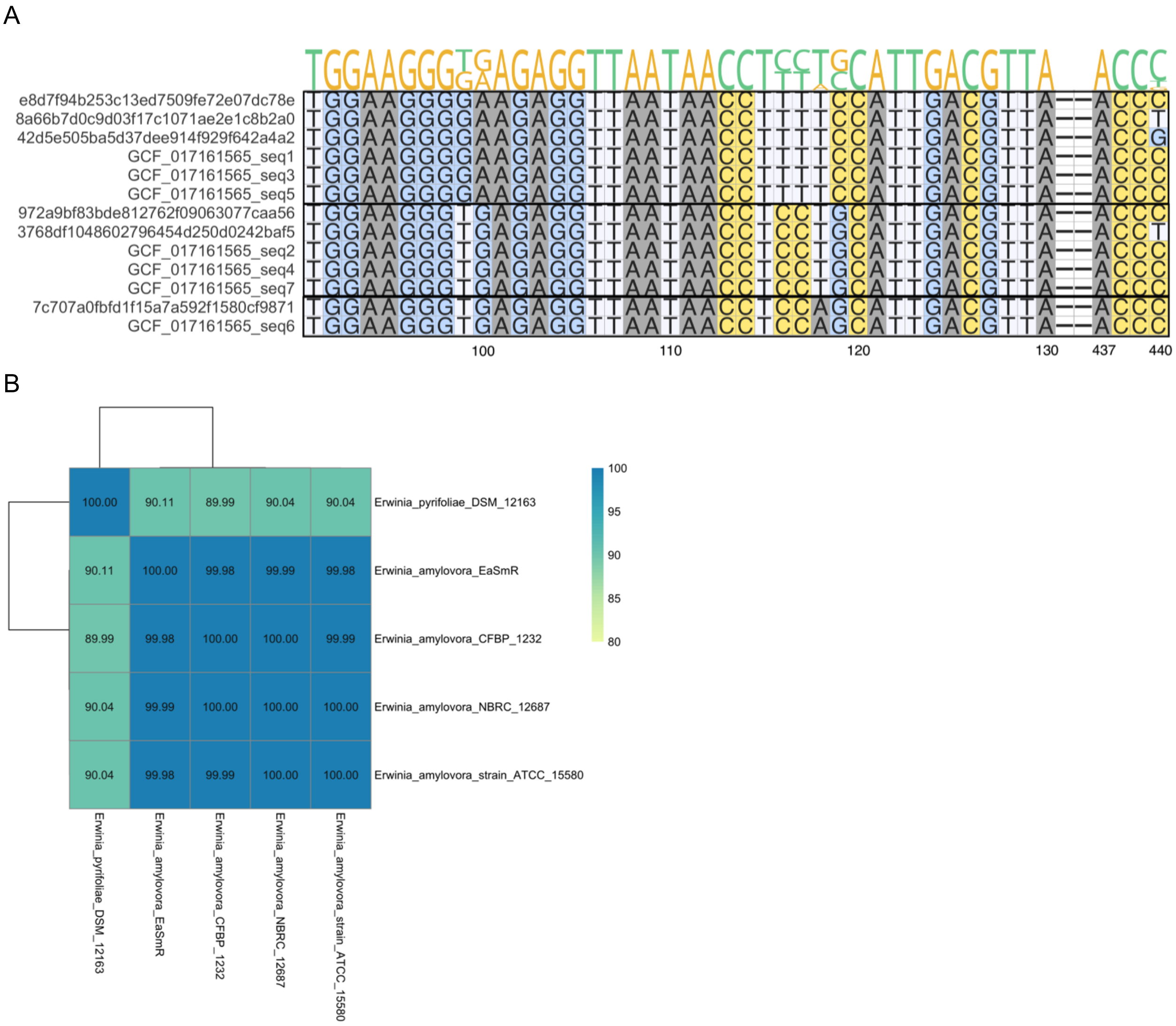

Figure S9: (A) Multiple sequence alignment of the six *Erwinia* ASVs detected in this study with the V3-V4 regions of the seven 16S rRNA copies of the best type-material hit from NCBI (*E. amylovora* st. ATCC 15580, assembly GCF_017161565.1). (B) ANI distances between the type-material genomes with the best hits to the *Erwinia* ASVs from this study. The ASVs were aligned against the core_nt database with search limited to type material through the BLAST website. ASVs aligned with *E. amylovora* st. ATCC 15580 (99.8-100% identity over all ASVs), *E. pyrifoiliae* DSM 12163 (98.64-99.32%), *E. amylovora* st. NBRC 12687 (98.64-98.86%), and *E. amylovora* st. CFBP 1232 (98.86%-98.18%). We added NCBI's representative genome *E. amylovora* st. EaSmR (GCF_043228865.1) to the ANI analysis despite not being a type-material.

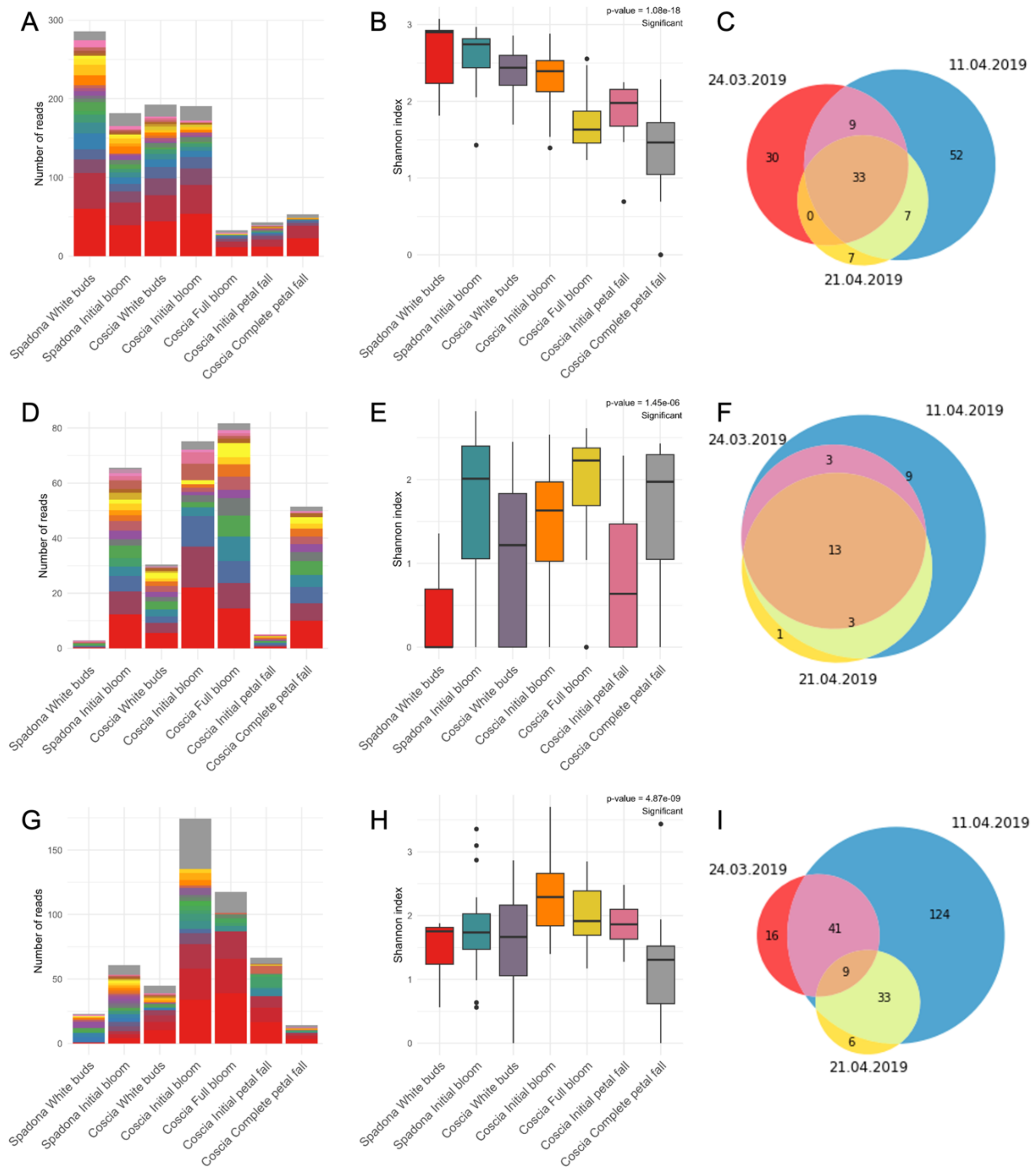

Figure S10: Left column: read counts, middle column: Shannon Index, right column: the number of shared ASVs between samples collected on the first collection date for Spadona (24.3), the first collection date for Coscia (11.4) and the last collection date (Coscia, 21.4). A-C: *Sphingomonas*; D-F: *Pantoea*; G-I: *Lactobacillus*. The datasets were rarefied as specified in the methods. The significance of the differences in the Shannon index values was tested using Kruskal-Walis tests. The p-value for *Sphingomonas* is $1.08*10^{-18}$; for *Pantoea* $1.45*10^{-6}$; and for *Lactobacillus*: $4.87*10^{-9}$.

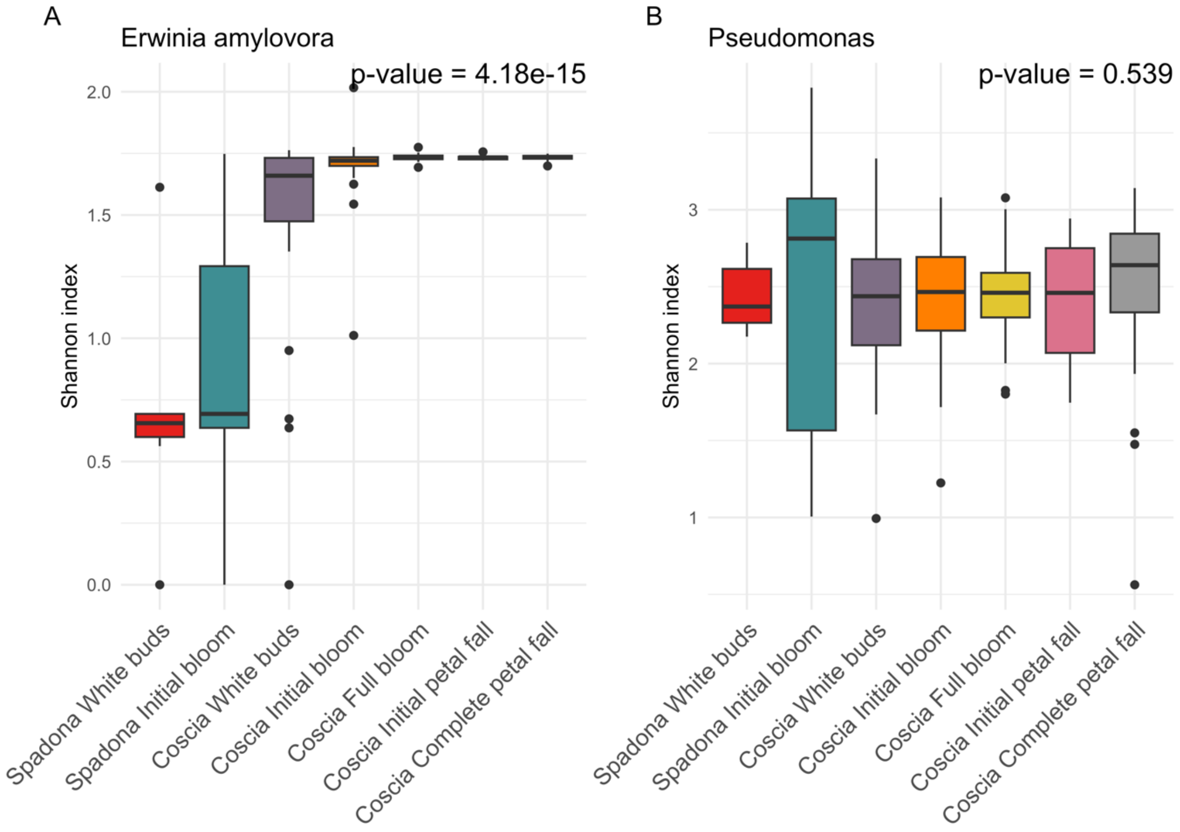

Figure S11: Shannon Index for populations of *Erwinia amylovora* (A) and *Pseudomonas* (B) by phenological stage and cultivar.
